# Impact of the Method of G6PD Deficiency Assessment on Genetic Association Studies of Malaria Susceptibility

**DOI:** 10.1371/journal.pone.0007246

**Published:** 2009-09-30

**Authors:** Marla K. Johnson, Tamara D. Clark, Denise Njama-Meya, Philip J. Rosenthal, Sunil Parikh

**Affiliations:** 1 Department of Medicine, San Francisco General Hospital, University of California San Francisco, San Francisco, California, United States of America; 2 Makerere University - UCSF Malaria Research Collaboration, Mulago Hospital, Kampala, Uganda; Walter and Eliza Hall Institute of Medical Research, Australia

## Abstract

**Background:**

Clinical association studies have yielded varied results regarding the impact of glucose-6-phosphate dehydrogenase (G6PD) deficiency upon susceptibility to malaria. Analyses have been complicated by varied methods used to diagnose G6PD deficiency.

**Methodology/Prinicipal Findings:**

We compared the association between uncomplicated malaria incidence and G6PD deficiency in a cohort of 601 Ugandan children using two different diagnostic methods, enzyme activity and G6PD genotype (G202A, the predominant East African allele). Although roughly the same percentage of males were identified as deficient using enzyme activity (12%) and genotype (14%), nearly 30% of males who were enzymatically deficient were wild-type at G202A. The number of deficient females was three-fold higher with assessment by genotype (21%) compared to enzyme activity (7%). Heterozygous females accounted for the majority (46/54) of children with a mutant genotype but normal enzyme activity. G6PD deficiency, as determined by G6PD enzyme activity, conferred a 52% (relative risk [RR] 0.48, 95% CI 0.31–0.75) reduced risk of uncomplicated malaria in females. In contrast, when G6PD deficiency was defined based on genotype, the protective association for females was no longer seen (RR = 0.99, 95% CI 0.70–1.39). Notably, restricting the analysis to those females who were both genotypically and enzymatically deficient, the association of deficiency and protection from uncomplicated malaria was again demonstrated in females, but not in males (RR = 0.57, 95% CI 0.37–0.88 for females).

**Conclusions/Significance:**

This study underscores the impact that the method of identifying G6PD deficient individuals has upon association studies of G6PD deficiency and uncomplicated malaria. We found that G6PD-deficient females were significantly protected against uncomplicated malaria, but this protection was only seen when G6PD deficiency is described using enzyme activity. These observations may help to explain the discrepancy in some published association studies involving G6PD deficiency and uncomplicated malaria.

## Introduction

Glucose-6-phosphate dehydrogenase (G6PD) catalyzes the first step of the pentose phosphate pathway, which converts glucose into pentose sugars for glycolysis and other biological reactions. The pentose phosphate pathway is the only source of reduced nicotinamide adenine dinucleotide phosphate (NADPH) in erythrocytes, and therefore the principal means by which erythrocytes counteract oxidative stress [1]. Deficiency in G6PD was first described in the 1950s as the cause of hemolysis in response to the antimalarial drug primaquine [2]. G6PD deficiency is the most common enzymopathy worldwide, affecting approximately 330 million people [3]. Deficiency is caused by mutations in the X-linked G6PD gene, in which more than 160 mutations have been identified [4], [5].

Malaria has exerted a tremendous selective pressure on the human genome, with an estimated 300–660 million cases of *P. falciparum* malaria, leading to approximately 1 million deaths each year [6], [7]. Many factors contribute to an individual's risk of malaria, including immunity from prior infections, age, genetic factors, use of preventive measures, and proximity to mosquito breeding sites [8], [9]. Coincident to the discovery of G6PD deficiency as the cause of antimalarial induced hemolysis, a strong geographical overlap was noted between the prevalence of G6PD deficiency and malaria endemicity [10], [11]. Based on this observation, it was hypothesized that G6PD deficiency had arisen as a protective factor against lethal malaria [10], [11]. Since that initial observation, dozens of clinical association studies have been performed, yielding varied results. Selected studies of severe disease showed a decrease in the risk of severe malaria in hemizygous males [12], [13] and either a reduced risk [13] or no association with risk in heterozygous females [12]. Studies of uncomplicated malaria have been more inconsistent, showing G6PD deficiency to be protective in heterozygous females [8], [13]–[15] or to have no effect on the incidence of uncomplicated malaria in either hemizygous males or heterozygous females [16]. Conversely, other studies showed an increase in the incidence of uncomplicated malaria in females heterozygous for G6PD deficiency [17], [18]. Definitive conclusions based on these results are difficult because studies differed in design, clinical phenotype assessed (severe or uncomplicated malaria), or methods to identify G6PD deficiency.

Most clinical screening tests measure the enzymatic formation of NADPH from NADP, such as the semiquantitative fluorescent spot test or the quantitative spectrophotometric test [19]–[22]. Many methodological issues exist with such tests, including the correct sample handling and storage, optimal anticoagulant for collection, and the use of appropriate control samples [23]. In addition, heterozygous females pose a particular challenge due to the phenomenon of variable X chromosome inactivation or lyonization. As a result, enzyme activity may vary depending on the proportion of normal and deficient cells which are inactivated in each individual [24]. G6PD deficiency may also be assessed by analysis for mutations in the G6PD gene [25]–[27]. The G6PD A- allele, which contains two mutations, G376A and G202A, is the most common G6PD deficiency variant in Africa, with a frequency of 0–25% [28]. The G376A mutation by itself results in the G6PD A allele, with 80% of the enzyme activity of wild type G6PD. When this mutation occurs in combination with G202A, the resultant G6PD A- allele produces an enzyme with approximately 12% the activity of wild-type G6PD [25], [26]. G202A nearly always occurs in the background of the mutation at position G376A [29]–[31]. Two other G6PD A- alleles, which account for 5% of this genotype, are due to mutations at nucleotide positions 680 or 968 in the setting of G376A [25]. In addition, recent evidence suggests that another low frequency mutation at position 542 may be present in West Africans [32]. As conflicting conclusions regarding malaria risk and G6PD deficiency may have been due to different detection methods, we characterized G6PD status by both enzymatic and genotypic assays in a cohort of Ugandan children and assessed correlations between the two measures and the risk of malaria.

## Materials and Methods

### Study site and recruitment of study participants

Participants for a randomized comparison of three combination antimalarial regimens were recruited from the Mulago III parish of Kampala, Uganda, as previously described [33]. In Kampala, malaria is mesoendemic, with a parasite prevalence of ∼20% in children ages two to nine years measured in 2004. Interim results of the clinical study have been published (isrctn.org identifier: ISRCTN37517549) [34]. Following a census, random households were selected to participate in the study, and 601 children, aged one to ten years, were recruited. The children were followed for two years beginning on April 1, 2005. Rainy seasons occur in March-May and September-November [33]. Subjects were not enrolled if they were severely malnourished, had a known adverse reaction to any study medication, had a known serious chronic disease, or were diagnosed with a life-threatening condition after a baseline laboratory screen.

### Ethics statement

This study was conducted according to the principles expressed in the Declaration of Helsinki. The clinical trial and this study were approved by the Uganda National Council of Science and Technology and Institutional Review Boards of Makerere University and the University of California, San Francisco. All patients' parents or guardians provided written informed consent for the collection of samples and subsequent analysis.

### Assessment of malarial incidence

All participants were asked to come to a study clinic for all their healthcare needs; the clinic was open seven days a week. Episodes of malaria were diagnosed by passive surveillance, and, in addition, subjects were assessed every 30 days. Subjects presenting to the clinic with fever (tympanic temperature ≥38.0°C) or history of fever in the previous 24 h had a thick blood smear assessed for parasites. If the smear was positive, the participant was treated for malaria, regardless of parasite density. Molecular genotyping was performed to distinguish new infections (incident events) from recrudescences (which were not considered incident events), following a step-wise algorithm including assessment of polymorphisms in the P. falciparum genes *msp1*, *msp2*, and four microsatellites [34], [35]. Treatment failures within three days of diagnosis were considered recrudescences and not counted as incident events. Further details regarding this cohort have been published [8].

### Laboratory techniques

Fresh whole blood samples were collected at enrollment and sent to a certified lab in Kampala for G6PD activity assessment. A single assessment was performed by estimating the rate of NADPH production from NADP using a commercially available quantitative spectrophotometric test (Randox Laboratories, Ardmore, UK, catalog number PD410). Strict adherence to manufacturer's protocol was followed including appropriate use of control samples. The manufacturer reports G6PD values as mU/10^9^ erythrocytes, and recommends a cutoff of 110 mU/10^9^ erythrocytes to differentiate normal from deficient G6PD activity.

For genotyping of G6PD, blood was collected on filter paper, and DNA was extracted using the QIAamp DNA Mini Kit (Qiagen, Valencia, CA). A specimen was unavailable for one individual, leaving a sample population of 600 individuals. Genotypes for the G6PD A- variant (G202A, rs1050828) were determined by PCR followed by restriction endonuclease digestion. PCR was performed using 2× PCR Master Mix (Fermentas, Glen Burnie, MD; 0.05 U/µl T*aq* Polymerase (recombinant), 4 mM MgCl_2_, and 0.4 mM of each dNTP), 0.2 µM of each primer (as described elsewhere [27]), 5% DMSO, and approximately 10 ng of DNA. Thermocycling was performed on a DNA Engine Dyan (MJ Research Inc., Waltham, MA). An initial denaturation at 94°C for 10 minutes was followed by 40 cycles of 94°C for 30 seconds, 68°C for 30 seconds, and 72°C for 30 seconds with a final extension of 72°C for 7 minutes. PCR products (10 µl) were incubated with 5 U of N*la*III at 37°C for three hours, and genotypes were determined by inspection of digestion products after 2.5% agarose gel electrophoresis.

In 110 randomly selected samples, genotypes for the G6PD A variant (G376A, rs1050829) were determined by PCR as above with primers as previously described [26]. Amplifications conditions were as above except for an annealing temperature of 55°C, followed by incubation with 5 U of *Fok*I at 37°C for two hours before electrophoresis and visual determination of genotypes.

Sixteen individuals who were found to be enzymatically deficient but were genotyped as wild-type for the 202 variant were sequenced to assess other alleles that define the G6PD A- variant (G680T and T968C). The amplification reactions were performed as described above, using primers described elsewhere [25]. Thermocycling conditions were as above, except for an annealing temperature of 65°C. Sequencing of PCR products was performed by the University of California San Francisco Genomics Core Facility.

### Statistical analysis

Statistical analysis was performed using Stata version 10 (Stata, College Station, TX). Predictor variables included age, sex, sickle cell trait, G6PD enzyme deficiency, G6PD genotype, bed net usage, materials used in household construction, household water source, and index of household wealth, household crowding, antimalarial treatment arm, and distance from potential mosquito breeding sites. Time-dependent covariates (age, bed net use, and calendar time) were evaluated at the precision of 1 day. Our outcome measure was malaria incidence, as measured by incident episodes of malaria per person-year at risk. Subjects who were in the cohort as of April 1, 2005, but were terminated from the study prior to completion of follow-up contributed person-time equivalent to the duration of time they spent in the study.

Univariate and multivariate analyses using generalized estimating equations with control for repeated measures in the same subject were used to estimate associations between predictor variables of interest and malaria. P<0.05 was considered statistically significant. Variables that were significant in univariate analysis were included in the multivariate model.

## Results

### Baseline G6PD levels

**Figure 1:**
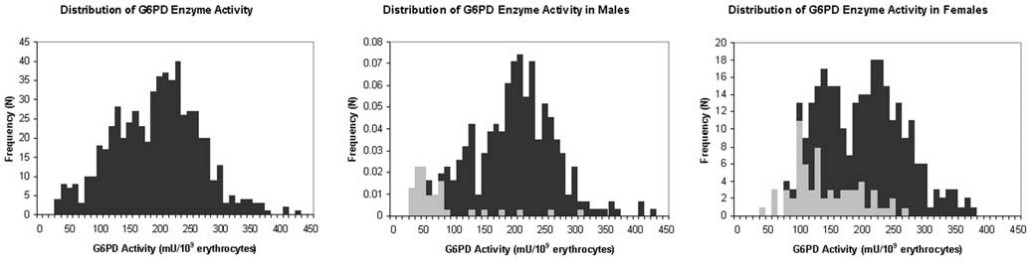
Distribution of G6PD enzyme activity (mU/10^9^ erythrocytes) in all individuals, males, and females. For the male and female distributions, G6PD A- genotype data are also displayed, with wild-type individuals represented by a dark gray bar and hemizygous, heterozygous, and homozygous individuals by a light gray bar.

A total of 600 individuals, consisting of 289 females and 311 males, were tested for G6PD enzyme activity (Figure 1). The shapes of the distributions differed, with the enzyme activity in males following a normal distribution and that in females more closely approximating a bimodal distribution. Based on the manufacturer's cutoff value of <110 mU/10^9^ erythrocytes, 20 females (6.9%) and 42 males (13.5%) had deficient G6PD enzyme activity.

### Genotyping for the A- Allele

Thirty-six males (11.6%) were hemizygous, 61 females (21.1%) were heterozygous and five females (1.7%) were homozygous for the G202A mutation. The allele frequency of G202A was 0.12 for both males and females. All genotypes were in Hardy-Weinberg equilibrium. To confirm that the G202A mutation occurred only in the background of the G376A mutation, we genotyped 110 randomly selected samples for the G6PD A variant. Of these 110 samples, 23 carried the G202 mutation, all of which also carried the G376A mutation. No individuals carried the G376A mutation in isolation.

### Correlation between G6PD genotype and phenotype

**Figure 2:**
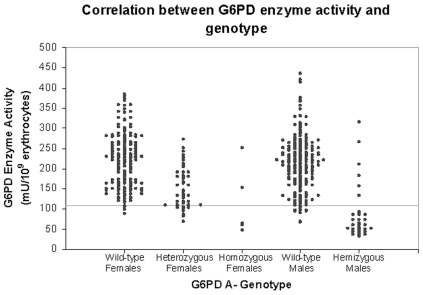
Dot plot showing the distribution of G6PD enzyme activity stratified by genotype.

Enzyme activity was stratified by gender and genotype (Figure 2). The median value of enzyme activity was 216 mU/10^9^ erythrocytes (Interquartile Range (IQR) = 80) for wild-type males and 61 mU/10^9^ erythrocytes (IQR = 38.5) for hemizygous males with the G202A allele. For females, the median value of enzyme activity was 225 mU/10^9^ erythrocytes (IQR = 101) for wild-type females, 135 mU/10^9^ erythrocytes (IQR = 77) for heterozygous females, and 63 mU/10^9^erythrocytes (IQR = 148) for homozygous G6PD A- females. Figure 1 also reveals that while the majority of genotypically-deficient females fell into the first peak of the bimodal enzyme distribution, a significant proportion of genotypically-deficient individuals had higher enzyme levels.

Only 6 of 269 (2%) males with normal G6PD enzyme activity carried the G6PD A- allele (Table 1). However, of the 269 females with normal enzyme activity, 48 (18%) were heterozygous and two (1%) were homozygous for the A- allele. Of the 42 males with deficient enzyme activity, 12 (29%) were wild-type at the A- allele. In the 20 females determined to have deficient enzyme activity, 4 (20%) were wild-type at the A- allele. Neither of the other mutations classified as G6PD A- variants (G680T and T968C) were found in the 16 individuals genotyped as wild-type for the G202A allele but found to be enzyme deficient.

**Table 1:** Correlation of Phenotype with Genotype by Gender.

Gender	Phenotype	N	Genotype	N	Enzyme Activity	
					Mean	Range
**Male**	Normal	269	Wild-type	263 (98%)	218	110–436
			Hemizygous	6 (2%)	211	132–316
	Deficient	42	Wild-type	12 (29%)	93	67–108
			Hemizygous	30 (71%)	59	32–93
**Female**	Normal	269	Wild-type	219 (81%)	222	116–385
			Heterozygous	48 (18%)	202	152–252
			Homozygous	2 (1%)	165	110–272
	Deficient	20	Wild-type	4 (20%)	100	88–107
			Heterozygous	13 (65%)	96	68–108
			Homozygous	3 (15%)	57	47–63

We assessed the sensitivity and specificity of the phenotypic test as compared to genetic analysis. For this analysis heterozygous females were classified as deficient. Using a cutoff of 110 mU/10∧9 erythrocytes to define G6PD deficiency in males, the test was 83.3% sensitive and 95.6% specific. In males, the test had a positive predictive value of 71.4% and a negative predictive value of 97.7%. In females, the test was 24.2% sensitive and 98.2% specific, with a positive predictive value of 80.0% and a negative predictive value of 81.4%.

### G6PD deficiency and the risk of malaria

We previously showed in this cohort that G6PD deficiency, as assessed solely by enzymatic assay, was associated with a significantly lower risk of uncomplicated malaria in females (relative risk (RR) = 0.48, 95% CI 0.31–0.75, p-value = 0.001) but not males (RR = 0.83, 95% CI 0.55–1.26, p-value = 0.39) [8]. With genetic results now available, we assessed the difference in risk of malaria between G6PD deficient individuals, as defined by enzymatic assay or genotype (Table 2). Notably, no association was seen between the incidence of uncomplicated malaria and the G6PD A- genotype in heterozygous and homozygous females (RR = 0.99, 95% CI 0.70–1.39, p-value = 0.95) or hemizygous males (RR = 0.79, 95% CI 0.52–1.23, p-value = 0.30; Table 2). As expected, the RRs for other predictor variables examined did not change between this study and our prior analysis.

**Table 2:** Association of predictor variables with the incidence of malaria.

Category	Group	New episodes of malaria	Person time (yrs)	Incidence of malaria	Multivariate analysis^a^		Multivariate analysis^b^	
					RR (95% CI)	p-value	RR (95% CI)	p-value
Age	Less than 6 years of age	305	338.1	0.90	1.0	-	1.0	-
	6 years or older	390	562.5	0.69	0.85 (0.70–1.04)	0.11	0.84 (0.69–1.02)	0.08
Sickle cell	AA	608	743.9	0.82	1.0	-	1.0	-
	AS	87	156.7	0.56	0.68 (0.52–0.90)	0.007	0.68 (0.52–0.90)	0.007
G6PD Activity	Normal female	290	391.8	0.74	1.0	-		
	Deficient female	11	36.5	0.30	0.48 (0.31–0.75)	0.001		
	Normal male	345	401.5	0.86	1.0	-		
	Deficient male	49	70.8	0.69	0.83 (0.55–1.26)	0.39		
G6PD Genotype	Wild-type female	228	318.5	0.72			1.0	-
	Homo/heterozygous female	75	110.2	0.68			0.99 (0.70–1.39)	0.95
	Wild-type male	352	411.7	0.85			1.0	-
	Hemizygous male	40	56.2	0.71			0.79 (0.52–1.23)	0.30
Bed net use	None	322	336.4	0.96	1.0	-	1.0	-
	Untreated	173	205.9	0.84	0.85 (0.68–1.07)	0.17	0.86 (0.69–1.08)	0.20
	Insecticide treated	200	358.3	0.56	0.51 (0.32–0.83)	0.006	0.52 (0.32–0.83)	0.007
Wealth index	1^st^ or 2^nd^ quartile	420	421.4	1.00	1.0	-	1.0	-
	3^rd^ quartile	155	234.4	0.66	0.83 (0.62–1.10)	0.20	0.83 (0.63–1.10)	0.20
	4^th^ quartile	120	244.8	0.49	0.77 (0.56–1.04)	0.09	0.78 (0.57–1.06)	0.11
Crowding	3 or less persons per room	268	471.5	0.57	1.0	-	1.0	-
	More than 3 persons per room	427	429.1	1.00	1.24 (0.98–1.57)	0.007	1.24 (0.98–1.57)	0.007
Distance from swamp	More than 200 meters from swamp	56	164.1	0.34	1.0	-	1.0	-
	101–200 meters from swamp	119	233.0	0.51	1.39 (0.91–2.10)	0.12	1.40 (0.92–2.13)	0.11
	1–100 meters from swamp	357	409.7	0.87	2.16 (1.51–3.10)	<0.001	2.21 (1.54–3.18)	<0.001
	Living in swamp	163	93.8	1.74	3.94 (2.61–5.97)	<0.001	4.07 (2.67–6.18)	<0.001

**NOTE** RR, relative risk.

a Previously published results from Table 1 [8], showing results of regression analysis using G6PD deficiency defined by enzyme assay.

b Repeat regression analysis using G6PD deficiency defined by genotype. Note that RR for other covariates remain constant.

As seen in both Table 1 and Figure 1, females defined as genotypically deficient (heterozygotes or homozygotes) were not uniformly identified as deficient by enzyme activity, a phenomenon explained largely by X chromosome inactivation. Thus, we repeated the association analysis, comparing the relative risk of uncomplicated malaria in females defined as both genotypically and enzymatically deficient to those females who were both genotypically wild-type and enzymatically normal. Indeed, females defined as deficient by both methods were protected from malaria compared with females defined as non-deficient by both methods (RR = 0.57, 95% CI 0.37–0.88). As before, males defined as deficient by genotype and enzyme activity did not appear significantly protected from uncomplicated malaria (RR 0.84, 95% CI 0.52–1.32).

Lastly, we assessed the impact of altering the cutoff value for the enzymatic test on our genetic association analysis (Table 3). At cutoff values between 90 (RR = 0.47, 95% CI 0.30–0.74, p-value = 0.001) and 120 (RR = 0.46, 95% CI 0.28–0.76, p-value = 0.002) mU/10^9^ erythrocytes, enzymatic G6PD deficiency was significantly associated with a decreased risk of uncomplicated malaria in females (Table 3). Considering cut-off values >120 mU/10^9^ erythrocytes, no association was seen between G6PD deficiency and uncomplicated malaria in females. Sample sizes for females with levels <90 were insufficient for meaningful statistical analysis. Notably, no significant association between G6PD deficiency and uncomplicated malaria was seen in males, even at a cut-off of <70 mU/10^9^ erythrocytes.

**Table 3:** Effect of G6PD activity cut-off criteria on the association analysis of G6PD status and malaria incidence.

G6PD Activity Cut-off Point (mU/10^9^ erythrocytes)	Enzymatically Deficient Female			Enzymatically Normal Male/Female		Enzymatically Deficient Male		
	N	RR (95% CI)	P-value	RR (95% CI)	P-value	N	RR (95% CI)	P-value
**<<<<<70**	4*	0.70 (0.42–1.20)	0.2	1	-	23	1.08 (0.70–1.68)	0.73
**<<<<<80**	4*	0.71 (0.42–1.20)	0.2	1	-	26	0.97 (0.61–1.53)	0.9
**<<<<<90**	8*	0.47 (0.30–0.74)	0.001	1	-	31	0.89 (0.57–1.37)	0.59
**<<<<<100**	10	0.58 (0.33–1.02)	0.061	1	-	39	0.82 (0.53–1.27)	0.37
**<<<<<110**	20	0.48 (0.31–0.75)	0.001	1	-	42	0.83 (0.55–1.26)	0.38
**<<<<<120**	33	0.46 (0.28–0.76)	0.002	1	-	51	0.87 (0.60–1.30)	0.53
**<<<<<130**	44	0.81 (0.52–1.26)	0.36	1	-	62	0.94 (0.65–1.33)	0.7
**<<<<<140**	61	0.93 (0.65–1.32)	0.69	1	-	73	0.88 (0.62–1.25)	0.49
**<<<<<150**	74	1.02 (0.74–1.42)	0.9	1	-	78	0.81 (0.57–1.16)	0.25

NOTE: N, represents the total number of individuals who have a G6PD activity level below the specified cut-off point; RR, relative risk.

NOTE: Table depicts how the relative risk of malaria varies depending on the cut-off point for determining G6PD deficiency by enzymatic assay.

* Sample sizes are too small for meaningful analysis of enzymatically deficient females in these categories.

## Discussion

Genetic association studies have provided inconsistent results on the relationship between G6PD deficiency and the risk of malaria, particularly uncomplicated malaria. Differences between studies include varied study designs, sample sizes, inclusion of other known genetic modifiers (ie. sickle cell), and differences in the malaria phenotype being assessed. A potential major source of discrepancy between studies was the method of screening for G6PD deficiency. We compared the results of the association between G6PD deficiency and malaria using both enzymatic and genetic assessments. We found that G6PD deficiency had no significant association with the incidence of uncomplicated malaria in males regardless of which assay was used. However, in females, when G6PD deficiency was defined by enzyme activity, deficiency was associated with a 52% reduced risk of uncomplicated malaria. This level of protection was comparable to that seen in individuals who use insecticide-treated bed nets (RR = 0.51) and somewhat greater than that conferred by sickle cell trait (RR = 0.68) [8]. Interestingly, the significant association between malaria incidence and G6PD phenotype in females was lost when G6PD status was defined by G6PD genotype (RR = 0.99 for heterozygous/homozygous females compared to wild-type females). However, in our cohort, out of 61 female heterozygotes, only 13 were defined as enzymatically deficient. Thus, we compared the risk of malaria in heterozygous/homozygous females who were also enzymatically deficient to the risk of malaria in those who were defined as normal by both assays. Notably, the “dually” deficient group was significantly protected from uncomplicated malaria. In other words, G6PD deficiency protected against uncomplicated malaria, but only in females when deficiency was measured enzymatically. It is possible that the varied results in prior association studies that have relied on genotypic testing may be explained due to the mosaic distribution of enzyme activity in heterozygous females.

As described earlier, the mosaic distribution of G6PD deficiency is due to variable X chromosome inactivation [36]. This characteristic explains, in large part, the lack of correlation between genotypic and enzymatic assays for the detection of G6PD deficiency. The overall prevalence of G6PD deficiency in males was similar between the genotypic (14% deficient) and enzymatic (12% deficient) assays. However, the assays did not correlate exactly, as almost 30% of males who were enzymatically deficient were wild-type for the G6PD A- allele. More strikingly, as noted above, we found a significant discrepancy between G6PD genotype and phenotype for females. Only 20 females (7%) were deficient by enzymatic assay, while 66 (23%) were genotyped as heterozygous or homozygous for the G6PD A- allele. Such findings may be of concern in instances where screening is performed to determine one's risk of drug-induced hemolysis [37]. Indeed, a recent trial of the antimalarial chlorproguanil-dapsone/artesunate found an unacceptably high rate of dapsone-induced hemolytic anemia in both homozygous and heterozygous females [38]. Given the low sensitivity of the enzymatic test for identifying heterozygous females, a significant number of females may be placed at risk of hemolysis if enzymatic screening is utilized in such settings [38].

To further explore the relationship between G6PD enzyme activity and the risk of malaria, we determined whether the degree of deficiency affected an individual's risk of malaria. With a cutoff of 90–120 mU/10^9^ erythrocytes, G6PD deficiency was consistently protective in females, with risk reductions between 42–54%. However, at higher cutoff values (<130 mU/10^9^ erythrocytes), protection from malaria was not seen. These results suggest that lower levels of enzyme activity are protective against malaria in females. Sample sizes were too small to meaningfully assess the association of levels of G6PD enzyme activity levels <90 mU/10^9^ erythrocytes in females. Notably, however, there was no evidence of a protective effect against uncomplicated malaria in the 23 males with the lowest G6PD activity levels (<70 mU/10^9^ erythrocytes). Prior studies have seen protection from malaria primarily in heterozygous females [14]. These findings have led to the hypothesis that in the face of a mosaic population of deficient erythrocytes, the malaria parasite is unable to efficiently adapt and produce its own G6PD enzyme [39].

Of 62 people with enzymatically determined G6PD deficiency, we were unable to identify a deficiency-causing variant in ∼25% (16/62) after assessing the three common G6PD A- variants found in African populations. Most notably, nearly 1 in 3 males who were enzymatically deficient, were wild-type at the G202A, G680T and T968C loci. It is likely that other mutations exist and that they may mediate altered enzyme activity in these individuals [32]. Full sequencing of the G6PD gene in our East African individuals is planned. Conversely, of the 538 individuals determined to have normal G6PD activity, a small minority (8/538) were homozygous or hemizygous for the G6PD A- variant. Normal enzyme level in these individuals might have been due to recent hemolysis, as reticulocytes have five times higher activity than older red blood cells [23]. We were not able to measure reticulocytes, but we did not detect any association between G6PD level and history of treatment for malaria within the two weeks preceding G6PD activity testing.

This study underscores the impact that the method of identifying G6PD deficient individuals has upon studies of G6PD deficiency and malaria. We found that G6PD-deficient females, but not males, were significantly protected against uncomplicated malaria, but this protection was only seen when G6PD deficiency was described using enzyme activity. The lack of association seen in heterozygous females was likely due to the mosaic population of erythrocytes, as protection was again seen when the analysis was restricted to females who were both heterozygous and enzymatically deficient. These observations may help to explain discrepancies in some published association studies involving G6PD deficiency and uncomplicated malaria. Ultimately, it may be that both methods have a role in the detection and characterization of G6PD deficiency. In the setting of association studies, whilst enzymatic assays seem to more closely approximate biologic function and correlate with protection, the addition of genetic testing may uncover novel disease causing variants and thereby aid in our understanding of this widespread enzymopathy.
